# Genetics of Unilateral and Bilateral Age-Related Macular Degeneration Severity Stages

**DOI:** 10.1371/journal.pone.0156778

**Published:** 2016-06-03

**Authors:** Tina Schick, Lebriz Altay, Eva Viehweger, Carel B. Hoyng, Anneke I. den Hollander, Moritz Felsch, Sascha Fauser

**Affiliations:** 1 Department of Ophthalmology, University Hospital of Cologne, Cologne, Germany; 2 Institute of Medical Statistics, Informatics and Epidemiology, University of Cologne, Cologne, Germany; 3 Department of Ophthalmology, Radboud University Medical Center, Nijmegen, The Netherlands; Massachusetts Eye & Ear Infirmary, Harvard Medical School, UNITED STATES

## Abstract

**Background:**

Age-related macular degeneration (AMD) is a common disease causing visual impairment and blindness. Various gene variants are strongly associated with late stage AMD, but little is known about the genetics of early forms of the disease. This study evaluated associations of genetic factors and different AMD stages depending on unilateral and bilateral disease severity.

**Methods:**

In this case-control study, participants were assigned to nine AMD severity stages based on the characteristics of each eye. 18 single nucleotide polymorphisms (SNPs) were genotyped and attempted to correlate with AMD severity stages by uni- and multivariate logistic regression analyses and trend analyses. Area under the receiver operating characteristic curves (AUC) were calculated.

**Results:**

Of 3444 individuals 1673 were controls, 379 had early AMD, 333 had intermediate AMD and 989 showed late AMD stages. With increasing severity of disease and bilateralism more SNPs with significant associations were found. Odds ratios, especially for the main risk polymorphisms in *ARMS2* (rs10490924) and *CFH* (rs1061170), gained with increasing disease severity and bilateralism (exemplarily: rs1061170: unilateral early AMD: OR = 1.18; bilateral early AMD: OR = 1.20; unilateral intermediate AMD: OR = 1.28; bilateral intermediate AMD: OR = 1.39, unilateral geographic atrophy (GA): OR = 1.50; bilateral GA: OR = 1.71). Trend analyses showed p<0.0001 for *ARMS2* (rs10490924) and for *CFH* (rs1061170), respectively. AUC of risk models for various AMD severity stages was lowest for unilateral early AMD (AUC = 0.629) and showed higher values in more severely and bilaterally affected individuals being highest for late AMD with GA in one eye and neovascular AMD in the other eye (AUC = 0.957).

**Conclusion:**

The association of known genetic risk factors with AMD became stronger with increasing disease severity, which also led to an increasing discriminative ability of AMD cases and controls. Genetic predisposition was also associated with the disease severity of the fellow-eye, highlighting the importance of both eyes in AMD patients.

## Introduction

In age-related macular degeneration (AMD), heritability plays a major role in disease development [1]. Several risk loci have been identified, but two loci are responsible for approximately 50% of the genetic risk: complement factor H (*CFH*) on chromosome 1q32 and age-related maculopathy susceptibility 2 (*ARMS2*)/HtrA serine peptidase 1 (*HTRA1*) on chromosome 10q26 [2].

In AMD, the phenotype of the early stages is characterized by drusen and pigmentary abnormalities. They usually cause little or no visual symptoms, but they are risk factors for progression to advanced AMD [3,4]. The advanced forms of AMD, namely geographic atrophy (GA) and choroidal neovascularisation (CNV), are the leading causes of visual impairment and blindness among the elderly [5]. These findings argue for the concept of a disease continuum starting with few alterations, progressing into an intermediate stage with large drusen, and finally resulting in the late-stage forms GA or neovascular AMD (nAMD). However, there are a lot of patients who develop nAMD with only few early signs of the disease.

The identification of associated genetic variants can help to detect mechanisms of this complex and heterogeneous condition. However, the majority of genetic studies were conducted with late-AMD cases [6]. Few studies included early forms of the disease or investigated progression factors [7–10]. Therefore, little is known about the role of genetic variants in early AMD or genetic factors driving the progression from early to late AMD. Twin studies indicated a lower heritability for early AMD of only about 35–53% [9]. This is significantly lower than the heritability for late AMD of up to 71% [11]. In a meta-analysis, only the two major risk genes *CFH* and *ARMS2/HTRA1* were associated with early AMD and the effect sizes were significantly lower than for late AMD [10]. However, the reason for these findings is not known. There are several possible explanations. There might be a clinical heterogeneity within the phenotype “early AMD” and some forms might be caused by other genetic factors. It is also possible that not all patients with signs of early AMD will progress to late stages because their retinal changes result from other factors. Another explanation could be different individual effect sizes for early and late AMD [12].

Hypothesizing, that there is not only a phenotypic, but also a genotypic disease continuum, we analyze the associations of 18 single nucleotide polymorphisms (SNPs) in individuals showing different AMD stages compared to controls. We also want to evaluate if genetic risk factors vary for different phenotypes. In this case-control study, we additionally want to determine the discrimination ability of genetic factors for unilateral and bilateral stages of early, intermediate, and late AMD.

## Methods

In our study, all participants with gradable images and available genetic analysis of the European Genetic Database (EUGENDA, www.eugenda.org) were included. EUGENDA is a large case-control database for AMD with participants from the areas around Cologne in Germany and Nijmegen in the Netherlands. Study participants were unrelated, self-described Caucasians, and at least 50 years of age. Controls were allowed to have other eye disease that did not restrict AMD grading. The study was performed in accordance with the tenets of the Declaration of Helsinki and the Medical Research Involving Human Subjects Act (WMO) and was approved by the local ethics committee of the University Hospitals in Cologne and Nijmegen. Written informed consent was obtained from all participants.

### Grading

AMD staging was performed by certified graders (TS and LE) according to the standard protocol of the Cologne Image Reading Center (CIRCL). Graders were blinded to the aims of this study. Grading of retinal images included stereo fundus photographs, fluorescein angiograms and SDOCTs. Controls were allowed to have only small drusen or pigmentary abnormalities with not more than 9 small drusen in the Early Treatment of Diabetic Retinopathy Study (ETDRS) grid. Control cases were additionally graded for changes of the outer retina in the whole SDOCT 6 mm x 4 mm volume scans (Spectralis SDOCT, Heidelberg Engineering, Heidelberg, Germany, performed by TS and EV). Eyes were termed as supercontrols, when both eyes showed an intact external limiting membrane, ellipsoid zone and retinal pigment epithelium (RPE)-Bruch´s complex.

Early AMD was classified by the presence of at least 10 small drusen (<63 μm) and pigmentary changes or the presence of 1–14 intermediate (63–124 μm) drusen in the ETDRS grid. Intermediate AMD was classified by the presence of ≥15 intermediate drusen or any large drusen (≥125 μm in diameter). Advanced AMD with geographic atrophy (GA) was defined as AMD with subfoveal atrophy of the RPE of at least 175μm diameter. Neovascular AMD (nAMD) was characterized by choroidal neovascularization (CNV) secondary to AMD. AMD staging was performed for both eyes separately and individuals were categorized in supercontrols, no AMD, unilateral early AMD, bilateral early AMD, unilateral intermediate AMD, bilateral intermediate AMD, unilateral GA, bilateral GA, unilateral nAMD, bilateral nAMD, late AMD mixed type. Late AMD mixed type was defined as CNV in one eye and GA in the other eye. In cases termed “unilateral”, the grading differed between both eyes. The classification was then based on the eye with the more severe AMD grading. (e.g. unilateral intermediate AMD could have no AMD or early AMD in the fellow-eye). In late AMD, no ranking of severity between GA and nAMD was made.

### Genotyping

Genomic DNA was extracted from peripheral blood samples using standard procedures. Eighteen SNPs in or near 13 AMD associated risk genes were chosen representing the major loci associated with AMD which are *CFH*, *ADAMTS9-AS2*, *COL8A1*, *CFI*, *C9*, *C2-CFB-SKIV2L*, *VEGFA*, *TNFRSF10A*, *TGFBR1*, *ARMS2-HTRA1*, *B3GALTL*, *RAD51B*, *LIPC*, *CETP*, *C3*, *APOE*, *SYN3-TIMP3*, *SLC16A8*, *COL4A3*, *PRLR-SPEF2*, *PILRB-PILRA*, *KMT2E-SRPK2*, *TRPM3*, *MIR6130-RORB*, *ABCA1*, *ARHGAP21*, *RDH5-CD63*, *ACAD10*, *CTRB2-CTRB1*, *TMEM97-VTN*, *NPLOC4-TSPAN10*, *CNN2*, *MMP9*, *C20orf85*. [13]. Genotyping of SNPs in the *ARMS2* (rs10490924), *CFH* (rs1061170, rs800292, rs12144939), *CFI* (rs10033900), *C3* (rs2230199, rs1047286), *CFB* (rs4151667, rs641153), *TIMP3* (rs9621532), *APOE* (rs2075650, rs4420638), *CETP* (rs3764261), *VEGFA* (rs943080), *TGFBR1* (rs334353), *SKIV2L* (rs429698), *RAD51B* (rs8017304) *and TNFRSF10A* (rs1327806) genes were carried out as previously described [14]. With an allele-specific PCR, a fluorescent signal is generated through the use of universal labeled primers, which can be quantified directly from microplates using standard plate readers. The assay is conducted in closed-tube format to reduce the potential for contamination to a minimum [14].

### Statistical analysis

Calculations were performed using SPSS software version 21.0 (IBM Software and Systems, Armonk, NY, USA). Associations between AMD severity stages and SNPs were calculated by univariate logistic regression analyses. For analysis, SNPs were categorized into “wild type”, the “heterozygous” risk variant, and the “homozygous” risk variant. All analyses were adjusted for age. Associations of other known risk factors (gender, smoking status, arterial hypertension and diabetes) were tested using Chi^2^-test, age was tested using an analysis of variance (ANOVA). Armitage’s trend test between the severity stages no AMD, unilateral early AMD, bilateral early AMD, unilateral intermediate AMD, bilateral intermediate AMD, unilateral late AMD and bilateral late AMD for each SNP (wild type / heterozygous vs. homozygous and wild type vs. heterozygous / homozygous) was performed using SAS software version 9.3. Power analysis was calculated with nQuery Advisor 7.0.

Odds ratios (OR) and 95% confidence intervals (CI) were calculated. P-values are two-sided and were analyzed with the Chi^2^-test. P-values <0.05 were considered statistically significant. For each severity stage, multivariate regression models were computed including all SNPs and age of participants. Variables were not categorized for better visualization and comparability of the results. The area under the receiver operating characteristic curve (AUC) was calculated to check the genetic discriminative ability between cases of different AMD stages and healthy controls.

Patients with unilateral nAMD were analyzed for associations with rs10490924 in *ARMS2* and rs1061170 in *CFH by* univariate regression analysis and models were calculated by multivariate regression including both SNPs and age. Patients were differentiated based on disease severity in the second eye with “no AMD”, “early AMD”, and “intermediate AMD”. Additionally, Armitage’s trend test was performed for these severity stages of the fellow eye and both SNPs.

## Results

In the analysis, we included 3444 participants. Demographics and grading results are shown in Table 1 and Table 2. For power calculation, Chi^2^-test was used comparing the patients with no AMD (n = 1673) to any other group (type I error = 5%). We would be able to detect odds ratios (e.g. wild type and heterozygous vs. homozygous) of 1.5 or greater with a power of 80% if the sample size in the other group reached at least n = 218.

**Table 1:** Demographics.

	No AMD	Early AMD	Intermediate AMD	Late AMD	p-value
Age (range)	69.0±7.9 (50–100)	72.1±8.0 (50–99)	73.8±9.0 (52–100)	76.9±8.3 (50–98)	<0.001*
Female Sex	956/1673 (57.1%)	226/379 (59.6%)	209/333 (62.8%)	636/1059 (60.1%)	0.18**
Smoking (ever)	914/1610 (56.8%)	188/357 (52.7%)	184/309 (59.5%)	539/886 (60.8%)	0.04**
Arterial Hypertension	644/1644 (39.2%)	138/372 (37.1%)	129/322 (40.1%)	344/967 (35.6%)	0.25**
Diabetes	126/1644 (7.7%)	35/372 (9.4%)	25/322 (7.8%)	113/967 (11.7%)	0.005**

*analyzed with ANOVA

**analyzed with Chi^2^-test

**Table 2:** Grading results.

AMD staging	n
supercontrols	435
**total no AMD**	**1673**
unilateral early AMD	240
bilateral early AMD	139
**total early AMD**	**379**
unilateral intermediate AMD	132
bilateral intermediate AMD	201
**total intermediate AMD**	**333**
unilateral neovascular AMD	431
bilateral neovascular AMD	462
unilateral geographic atrophy	41
bilateral geographic atrophy	70
mixed type	55
**total late AMD**	**989**
**total group**	**3444**

AMD = age-related macular degeneration, late AMD, mixed type shows neovascular AMD in one eye and geographic atrophy in the fellow-eye.

Distribution of SNPs and results of the trend analyses are summarized in Table 3 for rs10490924 in *ARMS2* and rs1061170 in *CFH*. Results of additional SNPs are presented in S1 Table.

**Table 3:** Distribution of single nucleotide polymorphisms rs10490924 in *ARMS2* and rs1061170 in *CFH* and trend test of AMD severity stages (data of other SNPs is shown in S1 Table).

AMD staging	*ARMS2* (rs10490924)	*CFH* (rs1061170)
no AMD	GG: 961 (57.9%)	TT: 646 (39.0%)
	GT: 606 (36.5%)	TC: 796 (48.1%)
	TT: 92 (5.5%)	CC: 214 (12.9%)
unilateral early AMD	GG: 119 (50.0%)	TT: 88 (37.4%)
	GT: 105 (44.1%)	TC: 116 (49.4%)
	TT: 14 (5.9%)	CC: 31 (13.2%)
bilateral early AMD	GG: 83 (60.6%)	TT: 42 (22.0%)
	GT: 43 (31.4%)	TC: 69 (46.9%)
	TT: 11 (8.0%)	CC: 28 (20.1%)
unilateral intermediate AMD	GG: 60 (45.5%)	TT: 47 (35.6%)
	GT: 56 (42.4%)	TC: 58 (43.9%)
	TT: 16 (12.1%)	CC: 27 (20.5%)
bilateral intermediate AMD	GG: 86 (43.2%)	TT: 44 (22.0%)
	GT: 85 (42.7%)	TC: 91 (45.5%)
	TT: 28 (14.1%)	CC: 65 (32.5%)
unilateral neovascular AMD	GG: 147 (34.6%)	TT: 81 (19.1%)
	GT: 180 (42.4%)	TC: 196 (46.2%)
	TT: 98 (23.1%)	CC: 147 (34.7%)
bilateral neovascular AMD	GG: 122 (26.8%)	TT: 90 (19.8%)
	GT: 210 (46.2%)	TC: 204 (44.8%)
	TT: 123 (27.0%)	CC: 161 35.4%)
unilateral geographic atrophy	GG: 16 (39.0%)	TT: 7 (17.1%)
	GT: 15 (36.6%)	TC: 23 (56.1%)
	TT: 10 (24.4%)	CC: 11 (26.8%)
bilateral geographic atrophy	GG: 21 (30.4%)	TT: 7 (10.0%)
	GT: 32 (46.4%)	TC: 39 (55.7%)
	TT: 16 (23.2%)	CC: 24 (34.3%)
late AMD mixed type	GG: 14 (25.5%)	TT: 9 (16.4%)
	GT: 26 (47.3%)	TC: 23 (41.8%)
	TT: 15 (27.3%)	CC: 23 (41.8%)
trend test (p-value)	<0.0001^a^ / <0.0001^b^	<0.0001^c^ / <0.0001^d^

AMD = age related macular degeneration

a: GG + GT vs. TT

b: GG vs. GT + TT

c: TT + TC vs. CC

d: TT vs. TC + CC

### Univariate regression analysis of SNPs

Univariate regression analysis of SNPs was performed for all AMD severity stages. Detailed results are outlined for main risk SNPs in *ARMS2* and *CFH* in Table 4 and for additional SNPs in S2 Table. Supercontrols showed no significant associations compared to subjects with no AMD. However, *ARMS2* and *CFH* rs1061170 showed lower OR in supercontrols but mostly without reaching significance. In general, with increasing severity and bilateralism more SNPs were associated with the disease and higher odds ratios were found.

**Table 4:** Univariate regression analysis for each severity stage with results for SNPs rs10490924 in *ARMS2* and rs1061170 in *CFH* (data of other SNPs is shown in S2 Table).

AMD staging	*ARMS2* (rs10490924)	*CFH* (rs1061170)
supercontrols	0.45, p = 0.03	0.65, p = 0.08
	0.52, p = 0.09	0.66, p = 0.08
unilateral early AMD	1.47, p = 0.008	1.08, p = 0.61
	1.34, p = 0.33	1.10, p = 0.68
bilateral early AMD	0.87, p = 0.49	1.40, p = 0.10
	1.55, p = 0.20	2.17, p = 0.003
unilateral intermediate AMD	1.59, p = 0.02	1.03, p = 0.90
	3.36, p = 9.44x10^-5^	1.80, p = 0.02
bilateral intermediate AMD	1.69, p = 0.002	1.70, p = 0.006
	4.14, p = 1.68x10^-8^	4.85, p = 3.56x10^-13^
unilateral neovascular AMD	2.08, p = 1.21x10^-8^	2.04, p = 1.35x10^-6^
	8.23, p = 1.00x10^-13^	5.97, p = 1.00x10^-13^
bilateral neovascular AMD	3.18, p = 1.01x10^-13^	2.07 p = 3.56x10^-6^
	14.86, p = 1.00x10^-13^	6.86 p = 1.00x10^-13^
unilateral geographic atrophy	1.60, p = 0.21	2.88, p = 0.02
	8.28, p = 1.60x10^-6^	5.52, p = 0.001
bilateral geographic atrophy	2.54, p = 0.001	4.76, p = 0.0002
	9.64, p = 8.77x10^-10^	11.97, p = 3.53x10^-8^
late AMD mixed type	3.42, p = 0.001	2.40, p = 0.04
	16.93, p = 5.21x10^-11^	9.93 p = 1.06x10^-7^

First line: heterozygous variant, second line: homozygous variant showing odds ratio and p-value. Analysis adjusted for age, reference: no AMD; SNPs = single nucleotide polymorphisms, AMD = age-related macular degeneration, interm. = intermediate, nAMD = neovascular AMD, GA = geographic atrophy.

### Multivariate regression model for each severity stage

In the multivariate regression model, all 18 SNPs were included and age was used as a covariate. Models were calculated for each AMD stage with “no AMD” as the reference variable. Increasing severity stages of AMD showed an almost continuous increase of odds ratios for *ARMS2* (rs10490924) and *CFH* (rs1061170) (Table 5). The influence of other SNPs was considerably lower, rarely reaching significant p-values (S3 Table). The AUC showed increasing values with increasing AMD severity. Bilateral disease showed a higher AUC than unilateral disease within each severity level. Highest genetic discrimination between controls and cases (AUC = 0.957) was observed for late AMD mixed type. Details are shown in Table 6.

**Table 5:** Multivariate regression model for each severity stage with results for SNPs rs10490924 in *ARMS2* and rs1061170 in *CFH* (data of other SNPs is shown in S3 Table).

AMD staging	*ARMS2* (rs10490924)	*CFH* (rs1061170)
unilateral early AMD	1.32 (1.02–1.72), p = 0.04	1.18 (0.86–1.63), p = 0.30
bilateral early AMD	1.08 (0.76–1.54), p = 0.68	1.20 (0.80–1.79), p = 0.38
unilateral intermediate AMD	1.97 (1.41–2.76), p = 7.91x10^-5^	1.28 (0.85–1.94), p = 0.23
bilateral intermediate AMD	2.83 (2.07–3.86), p = 6.06x10^-6^	1.39 (0.97–2.00), p = 0.08
unilateral neovascular AMD	3.01 (2.39–3.79), p = 6.24x10^-21^	1.80 (1.34–2.41), p = 9.36x10^-5^
bilateral neovascular AMD	4.76 (3.58–6.32), p = 6.49x10^-27^	1.75 (1.25–2.46), p = 6.06x10^-6^
unilateral geographic atrophy	4.86 (2.45–9.62), p = 5.98x10^-6^	1.50 (0.64–3.52), p = 0.36
bilateral geographic atrophy	3.36 (1.79–6.29), p = 0.0002	1.71 (0.78–3.72), p = 0.18
late AMD mixed type	4.27 (2.22–8.22), p = 1.36x10^-5^	2.45 (1.04–5.78), p = 0.04

Odds ratios and 95% Confidence intervals are shown, reference: no AMD.

**Table 6:** Area under the curve (AUC) of risk models in different AMD severity stages.

AMD staging	AUC	95% CI	p-value
unilateral early AMD	0.629	0.586–0.672	3.55x10^-8^
bilateral early AMD	0.669	0.611–0.727	2.85x10^-8^
unilateral intermediate AMD	0.789	0.747–0.831	1.70x10^-22^
bilateral intermediate AMD	0.831	0.792–0.870	1.45x10^-36^
unilateral neovascular AMD	0.835	0.805–0.865	9.86x10^-66^
bilateral neovascular AMD	0.917	0.896–0.937	8.93x10^-104^
unilateral geographic atrophy	0.902	0.826–0.979	3.70x10^-11^
bilateral geographic atrophy	0.933	0.893–0.974	7.15x10^-19^
late AMD mixed type	0.957	0.923–0.991	2.33x10^-22^

AMD = age-related macular degeneration, CI = confidence interval

### Severity levels of fellow-eyes in nAMD

Univariate regression analysis for rs10490924 in *ARMS2* and rs1061170 in *CFH* was performed for cases with nAMD in the study eye and different severity stages of the fellow-eye. With increasing severity of the fellow-eye, increasing ORs were found (Table 7). A regression model that included both SNPs revealed similar results (data not shown). The trend analysis showed p = 0.009 (GG + GT vs. TT) and p = 0.001 (GG vs. GT + TT) for rs10490924 in *ARMS2* and p = 0.01 (TT + TC vs. CC) and p = 0.03 (TT vs. TC + CC) for rs1061170 in *CFH*, indicating that the genetic associations increase with rising disease severity in the fellow-eye in unilateral nAMD.

**Table 7:** Univariate regression analysis for SNPs rs10490924 in ARMS2 and rs1061170 in CFH in patients with unilateral nAMD and different severity stages of the fellow-eye.

Staging of fellow-eye	*ARMS2* (rs10490924)	*CFH* (rs1061170)
No AMD	1.66 (1.19–2.32) p = 0.03	1.74 (1.27–2.39) p = 0.01
Early AMD	2.54 (1.87–3.46) p = 3.30x10^-9^	2.35 (1.73–3.19) p = 4.72x10^-8^
Intermediate AMD	3.45 (1.08–1.12) p = 4.00x10^-29^	3.01 (2.42–3.74) p = 2.82x10^-23^

Odds ratios and 95% confidence intervals are shown, analysis adjusted for age. reference: no AMD; SNP = single nucleotide polymorphism, AMD = age-related macular degeneration.

## Discussion

While genetic risk factors for late AMD are well established, less is known for early stages of the disease. In this study, we showed that genetic associations were stronger with increasing disease severity. Genetic discrimination between AMD and controls was higher for late stages of the disease and was also higher for bilateral compared to unilateral cases. For bilateral late AMD mixed type the discrimination was almost perfect.

It has been previously shown that genetic factors differ between early and late AMD [7,8]. Our results support these findings in more detail and suggest that there is a disease continuum from early to late AMD based on genetic factors.

We also found that only few loci were associated with early disease as opposed to late-stage disease. These results are in line with other studies showing differences in heritability [9,11] and effect size [10] between early and late AMD.

However, all AMD stages shared some risk variants. For example, risk variants in CFH and ARMS2 conferred a risk from early to late stages of the disease. In addition, it is possible that there are additional yet unknown unique risk variants driving the onset and the progression of AMD. Despite of large genome-wide association studies, these factors have not yet been detected. Therefore, other possibilities have to be taken into account. An explanation might be that different variants produce synergistic effects for progression of the disease, but the identification of those patterns would be very challenging. Another explanation could be a higher phenotypic and genetic heterogeneity of early AMD. Maybe, the time of onset of AMD is driven by different pathways in different subjects. This would lower the effect sizes of a particular risk variant on the population level despite similar effect sizes in individuals.

Additionally, there may be some forms of „early AMD”that do not progress to late AMD although their clinical appearance can not yet be distinguished from other, progressing forms of early AMD. On the other hand, studies on very old individuals indicate that the majority of patients will eventually develop late AMD [15].

In this study, only *CFH* variants showed an effect in early AMD in univariate analyses. The role of *CFH* in early AMD was also described in other reports [16,17]. In the ARED study, the involvement of the complement system was discussed to be associated with early drusen formation [18]. The effect of risk variants in other genes of the complement system such as *C3* and *CFB* as well as other pathways became significant only in intermediate and late AMD. This is also consistent with reports showing an involvement of these genes with late AMD [19,20].

Likewise, in our multivariate model, significant associations were only seen for the SNPs in *CFH* (rs800292 and rs12144939), *ARMS2* (rs10490924), and rs3764261 in the Cholesterylester transfer gene [21–23]. This again highlights the importance of CFH and ARMS2 in all stages of AMD.

We also analyzed very subtle age-related changes in SDOCT images. We compared persons without any retinal changes (“supercontrols”) with persons having minor abnormalities but not yet being classified as AMD (“no AMD”). Both groups showed no significant genetic difference. This goes in line with the findings of Ferris at al, where small drusen were called druplets to underline that they are normal aging changes without a higher risk for developing AMD [24]. However, although not reaching significance risk variants showed lower OR in supercontrols, which is suggestive that even these small changes are first signs of the disease and are at least partially driven by common AMD risk variants.

In this study, we demonstrated that the phenotype of both eyes and not only the worst affected may influence genetic susceptibility. We observed higher discrimination between cases and controls and higher genetic associations with more severe involvement of the fellow-eye. Previously published AMD prediction models include demographic, genetic and environmental factors for AMD, but the associations between phenotype and genotype are mostly based on the more severely affected eye [25]. However, this conventional case-control approach of association testing does not provide all the information of the complex disease.

Our study has several limitations: the design as a case-control study has no follow-up and therefore prediction models for AMD progression cannot be provided. Our results suggest an increased genetic influence of known genetic factors on late AMD compared to early AMD, but they are not able to explain the cause. Additionally, the effect of age on AMD development is enormous and despite an adjustment for age in our calculations there might be a remaining influence of age in our analysis. Further studies containing large numbers of cases with GA and late AMD mixed type are needed to validate our results.

Strength of our study was the use of multimodal imaging for AMD staging combining fundus photographs and SDOCT. This guaranteed a superior differentiation of phenotypes compared to the majority of genetic studies.

In summary, genetic risk variants primarily established for late AMD showed reduced effects on early stages of the disease. Associations of these variants with AMD became stronger with increasing disease severity, which also led to an increasing discriminative ability. Our results also underline the importance of the second eye in AMD patients because the genetic predisposition was also associated with the disease severity of the fellow-eye. Future studies are needed to identify the processes leading to the onset of early AMD and the progression to late AMD in order to develop treatments to prevent vision loss.

## Supporting Information

S1 Table Distribution of single nucleotide polymorphisms and trend test of AMD severity stages. (DOCX)

S2 Table Univariate regression analysis of SNPs in different AMD severity stages. (DOCX)

S3 Table Multivariate regression model of different AMD severity stages. (DOCX)

## References

[1] Seddon JM, Reynolds R, Maller J, Fagerness JA, Daly MJ, Rosner B (2009) Prediction model for prevalence and incidence of advanced age-related macular degeneration based on genetic, demographic, and environmental variables. Invest Ophthalmol Vis Sci 50: 2044–2053. 10.1167/iovs.08-3064 19117936 PMC3772781

[2] Fritsche LG, Chen W, Schu M, Yaspan BL, Yu Y, Thorleifsson G, et al (2013) Seven new loci associated with age-related macular degeneration. Nat Genet 45: 433–439, 439e431-432. 10.1038/ng.2578 23455636 PMC3739472

[3] de Jong PT (2006) Age-related macular degeneration. N Engl J Med 355: 1474–1485. 17021323 10.1056/NEJMra062326

[4] Ferris FL, Davis MD, Clemons TE, Lee LY, Chew EY, Lindblad AS, et al (2005) A simplified severity scale for age-related macular degeneration: AREDS Report No. 18. Arch Ophthalmol 123: 1570–1574. 16286620 10.1001/archopht.123.11.1570 PMC1473206

[5] Bressler NM (2004) Age-related macular degeneration is the leading cause of blindness. JAMA 291: 1900–1901. 15108691 10.1001/jama.291.15.1900

[6] Fritsche LG, Fariss RN, Stambolian D, Abecasis GR, Curcio CA, Swaroop A (2014) Age-related macular degeneration: genetics and biology coming together. Annu Rev Genomics Hum Genet 15: 151–171. 10.1146/annurev-genom-090413-025610 24773320 PMC4217162

[7] Chakravarthy U, McKay GJ, de Jong PT, Rahu M, Seland J, Soubrane G, et al (2013) ARMS2 increases the risk of early and late age-related macular degeneration in the European Eye Study. Ophthalmology 120: 342–348. 10.1016/j.ophtha.2012.08.004 23098369

[8] Farwick A, Dasch B, Weber BH, Pauleikhoff D, Stoll M, Hense HW (2009) Variations in five genes and the severity of age-related macular degeneration: results from the Muenster aging and retina study. Eye (Lond) 23: 2238–2244. 19169232 10.1038/eye.2008.426

[9] Hammond CJ, Webster AR, Snieder H, Bird AC, Gilbert CE, Spector TD (2002) Genetic influence on early age-related maculopathy: a twin study. Ophthalmology 109: 730–736. 11927430 10.1016/s0161-6420(01)01049-1

[10] Holliday EG, Smith AV, Cornes BK, Buitendijk GH, Jensen RA, Sim X, et al (2013) Insights into the genetic architecture of early stage age-related macular degeneration: a genome-wide association study meta-analysis. PLoS One 8: e53830 10.1371/journal.pone.0053830 23326517 PMC3543264

[11] Seddon JM, Cote J, Page WF, Aggen SH, Neale MC (2005) The US twin study of age-related macular degeneration: relative roles of genetic and environmental influences. Arch Ophthalmol 123: 321–327. 15767473 10.1001/archopht.123.3.321

[12] Grassmann F, Fauser S, Weber BH (2015) The genetics of age-related macular degeneration (AMD)—Novel targets for designing treatment options? Eur J Pharm Biopharm 95: 194–202. 10.1016/j.ejpb.2015.04.039 25986585

[13] Fritsche LG, Igl W, Bailey JN, Grassmann F, Sengupta S, Bragg-Gresham JL, et al (2016) A large genome-wide association study of age-related macular degeneration highlights contributions of rare and common variants. Nat Genet 48: 134–143. 10.1038/ng.3448 26691988 PMC4745342

[14] Hawkins JR, Khripin Y, Valdes AM, Weaver TA (2002) Miniaturized sealed-tube allele-specific PCR. Hum Mutat 19: 543–553. 11968087 10.1002/humu.10060

[15] Hermann M, Caramoy A, Schroder S, Droge K, Kirchhof B, Fauser S (2012) Prevalence of age-related macular degeneration in persons aged 90 years and older in Cologne. Acta Ophthalmol 90: e500–501. 10.1111/j.1755-3768.2011.02347.x 22268633

[16] Delcourt C, Delyfer MN, Rougier MB, Amouyel P, Colin J, Le Goff M, et al (2011) Associations of complement factor H and smoking with early age-related macular degeneration: the ALIENOR study. Invest Ophthalmol Vis Sci 52: 5955–5962. 10.1167/iovs.10-6235 21642625

[17] Dietzel M, Pauleikhoff D, Arning A, Heimes B, Lommatzsch A, Stoll M, et al (2014) The contribution of genetic factors to phenotype and progression of drusen in early age-related macular degeneration. Graefes Arch Clin Exp Ophthalmol 252: 1273–1281. 10.1007/s00417-014-2690-7 24970616

[18] Yu Y, Reynolds R, Fagerness J, Rosner B, Daly MJ, Seddon JM (2011) Association of variants in the LIPC and ABCA1 genes with intermediate and large drusen and advanced age-related macular degeneration. Invest Ophthalmol Vis Sci 52: 4663–4670. 10.1167/iovs.10-7070 21447678 PMC3175969

[19] Ardeljan D, Meyerle CB, Agron E, Wang JJ, Mitchell P, Chew EY, et al (2013) Influence of TIMP3/SYN3 polymorphisms on the phenotypic presentation of age-related macular degeneration. Eur J Hum Genet 21: 1152–1157. 10.1038/ejhg.2013.14 23422939 PMC3778351

[20] Seddon JM, Silver RE, Kwong M, Rosner B (2015) Risk Prediction for Progression of Macular Degeneration: 10 Common and Rare Genetic Variants, Demographic, Environmental, and Macular Covariates. Invest Ophthalmol Vis Sci 56: 2192–2202. 10.1167/iovs.14-15841 25655794 PMC4405097

[21] Ng TK, Chen LJ, Liu DT, Tam PO, Chan WM, Liu K, et al (2008) Multiple gene polymorphisms in the complement factor h gene are associated with exudative age-related macular degeneration in chinese. Invest Ophthalmol Vis Sci 49: 3312–3317. 10.1167/iovs.07-1517 18421087

[22] Ristau T, Paun C, Ersoy L, Hahn M, Lechanteur Y, Hoyng C, et al (2014) Impact of the common genetic associations of age-related macular degeneration upon systemic complement component C3d levels. PLoS One 9: e93459 10.1371/journal.pone.0093459 24675670 PMC3968152

[23] Wang YF, Han Y, Zhang R, Qin L, Wang MX, Ma L (2015) CETP/LPL/LIPC gene polymorphisms and susceptibility to age-related macular degeneration. Sci Rep 5: 15711 10.1038/srep15711 26503844 PMC4621603

[24] Ferris FL 3rd, Wilkinson CP, Bird A, Chakravarthy U, Chew E, Csaky K, et al (2013) Clinical classification of age-related macular degeneration. Ophthalmology 120: 844–851. 10.1016/j.ophtha.2012.10.036 23332590 PMC11551519

[25] Buitendijk GH, Rochtchina E, Myers C, van Duijn CM, Lee KE, Klein BE, et al (2013) Prediction of age-related macular degeneration in the general population: the Three Continent AMD Consortium. Ophthalmology 120: 2644–2655. 10.1016/j.ophtha.2013.07.053 24120328 PMC3986722

