# Supplementary material for: Genetics of Unilateral and Bilateral Age-Related Macular Degeneration Severity Stages
Source: PLoS One. 2016 Jun 3;11(6):e0156778. doi: 10.1371/journal.pone.0156778 (PMC4892556; doi:10.1371/journal.pone.0156778)
Supplement: S1 Table — (DOCX) [file pone.0156778.s001.docx]

S1 Table: Distribution of single nucleotide polymorphisms and trend test of AMD severity stages

| **SNP** | | **no AMD** | **unilateral early AMD** | **bilateral early AMD** | **unilateral interm.AMD** | **bilateral interm. AMD** | **unilateral nAMD** | **bilateral nAMD** | **unilateral GA** | **bilateral GA** | **late AMD mixed type** | **trend test* (p-values)** |
| --- | --- | --- | --- | --- | --- | --- | --- | --- | --- | --- | --- | --- |
| CFH rs800292 | GG | 937 (56.5%) | 129 (53.8%) | 88 (63.8%) | 68 (51.5%) | 141 (70.1%) | 318 (74.1%) | 352 (76.9%) | 26 (63.4%) | 53 (76.8%) | 42 (77.8%) | <0.0001 |
|  | GA | 633 (38.2%) | 90 (37.5%) | 41 (29.7%) | 59 (44.7%) | 52 (25.9%) | 98 (22.8%) | 97 (21.2%) | 13 (31.7%) | 15 (21.7%) | 12 (22.2%) | <0.0001 |
|  | AA | 87 (5.3%) | 21 (8.8%) | 9 (6.5%) | 5 (3.8%) | 8 (4.0%) | 13 (3.0%) | 9 (2.0%) | 2 (4.9%) | 1 (1.4%) | 0 (0.0%) |  |
| CFH rs12144939 | GG | 1058 (63.6%) | 154 (64.2%) | 100 (71.9%) | 96 (72.7%) | 169 (84.1%) | 340 (80.0%) | 375 (81.7%) | 34 (82.9%) | 60 (85.7%) | 45 (81.8%) | 0.0001 |
|  | GT | 536 (32.2%) | 78 (32.5%) | 36 (25.9%) | 32 (24.2%) | 27 (13.4%) | 77 (18.1%) | 78 (17.0%) | 7 (17.1%) | 9 (12.9%) | 9 (16.4%) | <0.0001 |
|  | TT | 69 (4.1%) | 8 (3.3%) | 3 (2.2%) | 4 (3.0%) | 5 (2.5%) | 8 (1.9%) | 6 (1.3%) | 0 (0.0%) | 1 (1.4%) | 1 (1.8%) |  |
| C3 rs1047286 | GG | 824 (63.7%) | 115 (61.2%) | 64 (62.1%) | 66 (62.9%) | 80 (55.9%) | 182 (65.0%) | 161 (54.6%) | 15 (62.5%) | 20 (52.6%) | 22 (54.2%) | <0.0001 |
|  | GA | 429 (33.2%) | 63 (33.5%) | 35 (34.0%) | 29 (27.6%) | 54 (37.8%) | 84 (30.0%) | 112 (38.0%) | 7 (29.2%) | 12 (31.6%) | 18 (42.9%) | 0.01 |
|  | AA | 40 (3.1%) | 10 (5.3%) | 4 (3.9%) | 10 (9.5%) | 9 (6.3%) | 14 (5.0%) | 22 (7.5%) | 2 (8.3%) | 6 (15.8%) | 2 (4.8%) |  |
| C3 rs2230199 | CC | 812 (62.8%) | 114 (60.6%) | 65 (63.1%) | 67 (63.8%) | 80 (55.9%) | 177 (63.2%) | 154 (52.2%) | 15 (62.5%) | 19 (50.0%) | 21 (50.0%) | <0.0001 |
|  | CG | 437 (33.8%) | 60 (31.9%) | 34 (33.0%) | 27 (25.7%) | 54 (37.8%) | 90 (32.1%) | 117 (39.7%) | 7 (29.2%) | 13 (34.2%) | 16 (38.1%) | 0.003 |
|  | GG | 44 (3.4%) | 14 (7.4%) | 4 (3.9%) | 11 (10.5%) | 9 (6.3%) | 13 (4.6%) | 24 (8.1%) | 2 (8.3%) | 6 (15.8%) | 5 (11.9%) |  |
| CFB rs4151667 | TT | 1519 (91.5%) | 211 (87.9%) | 125 (89.8%) | 126 (95.5%) | 190 (95.0%) | 407 (95.3%) | 437 (94.8%) | 39 (95.1%) | 68 (97.1%) | 54 (98.2%) | 0.87 |
|  | TA | 141 (8.5%) | 29 (12.1%) | 14 (10.1%) | 6 (5.0%) | 10 (12.1%) | 19 (4.4%) | 24 (5.2%) | 2 (4.9%) | 2 (2.9%) | 1 (1.8%) | <0.0001 |
|  | AA | 1 (0.1%) | 0 (0.0%) | 0 (0.0%) | 0 (0.0%) | 0 (0.0%) | 1 (0.2%) | 0 (0.0%) | 0 (0.0%) | 0 (0.0%) | 0 (0.0%) |  |
| CFB rs641153 | GG | 1392 (83.7%) | 199 (82.9%) | 122 (87.8%) | 116 (87.9%) | 185 (92.0%) | 386 (90.2%) | 419 (91.3%) | 34 (82.9%) | 66 (94.3%) | 52 (94.5%) | 0.22 |
|  | GA | 263 (15.8%) | 40 (16.7%) | 17 (12.2%) | 16 (12.1%) | 16 (8.0%) | 39 (9.1%) | 40 (8.7%) | 7 (17.1%) | 4 (5.7%) | 3 (5.5%) | <0.0001 |
|  | AA | 8 (0.5%) | 1(0.4%) | 0 (0.0%) | 0 (0.0%) | 0 (0.0%) | 3 (0.7%) | 0 (0.0%) | 0 (0.0%) | 0 (0.0%) | 0 (0.0%) |  |
| CFI rs10033900 | CC | 437 (26.2%) | 69 (29.4%) | 36 (26.3%) | 33 (25.0%) | 51 (25.8%) | 112 (26.3%) | 119 (25.9%) | 5 (12.5%) | 15 (21.4%) | 8 (15.1%) | 0.04 |
|  | CT | 864 (51.9%) | 120 (51.1%) | 64 (46.7%) | 64 (48.5%) | 96 (48.5%) | 215 (50.5%) | 218 (47.5%) | 26 (65.0%) | 38 (54.3%) | 34 (64.2%) | 0.29 |
|  | TT | 364 (21.9%) | 46 (19.6%) | 37 (27.0%) | 35 (26.5%) | 51 (25.8%) | 99 (23.2%) | 122 (26.6%) | 9 (22.5%) | 17 (24.3%) | 11 (20.8%) |  |
| CETP rs3764261 | GG | 799 (48.0%) | 107 (44.8%) | 68 (48.9%) | 53 (40.2%) | 81 (40.5%) | 185 (43.2%) | 182 (39.7%) | 18 (43.9%) | 28 (40.0%) | 24 (43.6%) | 0.11 |
|  | GT | 681 (40.9%) | 100 (41.8%) | 54 (38.8%) | 57 (43.2%) | 85 (42.5%) | 188 (43.9%) | 216 (47.2%) | 20 (48.8%) | 35 (50.0%) | 23 (41.8%) | 0.0003 |
|  | TT | 184 (11.1%) | 32 (13.4%) | 17 (12.2%) | 22 (16.7%) | 34 (17.0%) | 55 (12.9%) | 60 (13.1%) | 3 (7.3%) | 7 (10.0%) | 8 (14.5%) |  |
| TIMP3 rs9621532 | AA | 1504 (90.2%) | 219 (91.6%) | 126 (90.6%) | 124 (93.9%) | 179 (89.1%) | 400 (93.0%) | 438 (95.0%) | 37 (90.2%) | 58 (82.9%) | 54 (98.2%) | 0.42 |
|  | AC | 158 (9.5%) | 20 (8.4%) | 13 (9.4%) | 8 (6.1%) | 22 (10.9%) | 30 (7.0%) | 22 (4.8%) | 4 (9.8%) | 11 (15.7%) | 1 (1.8%) | 0.006 |
|  | CC | 6 (0.4%) | 0 (0.0%) | 0 (0.0%) | 0 (0.0%) | 0 (0.0%) | 0 (0.0%) | 1 (0.2%) | 0 (0.0%) | 1 (1.4%) | 0 (0.0%) |  |
| APOE rs2075650 | AA | 1247 (74.8%) | 172 (71.7%) | 103 (74.1%) | 108 (83.1%) | 158 (78.6%) | 355 (82.8%) | 352 (76.4%) | 32 (78.0%) | 59 (84.3%) | 47 (85.5%) | 0.90 |
|  | AG | 393 (23.6%) | 65 (27.1%) | 34 (24.5%) | 21 (16.2%) | 41 (20.4%) | 71 (16.6%) | 97 (21.0%) | 9 (22.0%) | 9 (12.9%) | 8 (14.5%) | 0.0009 |
|  | GG | 27 (1.6%) | 3 (1.3%) | 2 (1.4%) | 1 (0.8%) | 2 (1.0%) | 3 (0.6%) | 12 (2.6%) | 0 (0.0%) | 2 (2.6%) | 0 (0.0%) |  |
| APOE rs4420638 | AA | 1129 (68.3%) | 167 (70.5%) | 101 (72.7%) | 103 (79.2%) | 147 (73.5%) | 317 (73.7%) | 325 (71.0%) | 29 (70.7%) | 55 (78.6%) | 39 (70.9%) | 0.13 |
|  | AG | 474 (28.7%) | 66 (27.8%) | 36 (25.9%) | 25 (19.2%) | 49 (24.5%) | 108 (25.1%) | 122 (26.6%) | 11 (26.8%) | 12 (17.1%) | 15 (27.3%) | 0.01 |
|  | GG | 50 (3.0%) | 4 (1.7%) | 2 (1.4%) | 2 (1.5%) | 4 (2.0%) | 5 (1.2%) | 11 (2.4%) | 1 (2.4%) | 3 (4.3%) | 1 (1.8%) |  |
| TGFBR1rs334353 | TT | 966 (57.9%) | 129 (53.8%) | 77 (55.4%) | 72 (54.5%) | 126 (63.0%) | 244 (57.0%) | 284 (61.7%) | 23 (56.1%) | 43 (61.4%) | 33 (60.0%) | 0.02 |
|  | TG | 591 (35.5%) | 96 (40.0%) | 57 (41.0%) | 50 (37.9%) | 66 (33.0%) | 164 (38.3%) | 158 (34.3%) | 15 (36.6%) | 21 (30.0%) | 21 (38.2%) | 0.19 |
|  | GG | 110 (6.6%) | 15 (6.3%) | 5 (3.6%) | 10 (7.6%) | 8 (4.0%) | 20 (4.7%) | 18 (3.9%) | 3 (7.3%) | 6 (8.6%) | 1 (1.8%) |  |
| SKIV2L rs429608 | GG | 1246 (75.2%) | 168 (70.0%) | 105 (75.5%) | 107 (81.7%) | 172 (86.0%) | 362 (84.4%) | 386 (84.3%) | 31 (75.6%) | 62 (88.6%) | 50 (90.9%) | 0.04 |
|  | GA | 385 (23.2%) | 69 (28.7%) | 32 (23.0%) | 21 (16.0%) | 26 (13.0%) | 61 (14.2%) | 70 (15.3%) | 10 (24.4%) | 8 (11.4%) | 5 (9.1%) | <0.0001 |
|  | AA | 27 (1.6%) | 3 (1.3%) | 2 (1.4%) | 3 (2.3%) | 2 (1.0%) | 6 (1.4%) | 2 (0.4%) | 0 (0.0%) | 0 (0.0%) | 0 (0.0%) |  |
| VEGFA rs943080 | TT | 436 (26.1%) | 61 (25.4%) | 40 (28.8%) | 29 (22.0%) | 57 (28.4%) | 127 (29.5%) | 141 (30.7%) | 6 (14.6%) | 21 (30.0%) | 19 (34.5%) | 0.12 |
|  | TC | 846 (50.7%) | 125 (52.1%) | 71 (51.1%) | 58 (43.9%) | 110 (54.7%) | 208 (48.4%) | 225 (49.0%) | 25 (61.0%) | 37 (52.9%) | 25 (45.5%) | 0.03 |
|  | CC | 387 (23.2%) | 54 (22.5%) | 28 (20.1%) | 45 (34.1%) | 34 (16.9%) | 95 (22.1%) | 93 (20.3%) | 10 (24.4%) | 12 (17.1%) | 11 (20.0%) |  |
| RAD51B rs8017304 | AA | 639 (38.4%) | 89 (37.1%) | 54 (38.8%) | 52 (39.4%) | 83 (41.3%) | 179 (41.9%) | 209 (45.3%) | 15 (36.6%) | 32 (45.7%) | 26 (47.3%) | 0.004 |
|  | AG | 771 (46.4%) | 123 (51.2%) | 64 (46.0%) | 61 (46.2%) | 84 (41.8%) | 200 (46.8%) | 211 (45.8%) | 22 (53.7%) | 28 (40.0%) | 19 (34.5%) | 0.003 |
|  | GG | 252 (15.2%) | 28 (11.7%) | 21 (15.1%) | 19 (14.4%) | 34 (16.9%) | 48 (11.2%) | 41 (8.9%) | 4 (9.8%) | 10 (14.3%) | 10 (18.2%) |  |
| TNFRSF10A rs1327806 | TT | 444 (26.7%) | 70 (29.2%) | 31 (22.8%) | 41 (31.1%) | 56 (28.3%) | 117 (27.3%) | 125 (27.4%) | 7 (17.5%) | 28 (40.0%) | 25 (46.3%) | 0.007 |
|  | TG | 822 (49.3%) | 120 (50.0%) | 72 (52.9%) | 63 (47.7%) | 107 (54.0%) | 230 (53.7%) | 240 (52.6%) | 16 (40.0%) | 31 (44.3%) | 20 (37.0%) | 0.16 |
|  | GG | 400 (24.0%) | 50 (20.8%) | 33 (24.3%) | 28 (21.2%) | 35 (17.7%) | 81 (18.9%) | 91 (20.0%) | 17 (42.5%) | 11 (15.7%) | 9 (16.7%) |  |

AMD = age-related macular degeneration, interm. = intermediate, nAMD = neovascular AMD, GA = geographic atrophy; *first line: wild type / heterozygous vs. homozygous; second line: wild type vs. heterozygous / homozygous).
