# Supplementary material for: Genetics of Unilateral and Bilateral Age-Related Macular Degeneration Severity Stages
Source: PLoS One. 2016 Jun 3;11(6):e0156778. doi: 10.1371/journal.pone.0156778 (PMC4892556; doi:10.1371/journal.pone.0156778)
Supplement: S2 Table — (DOCX) [file pone.0156778.s002.docx]

S2 Table: Univariate regression analysis of SNPs in different AMD severity stages

| **SNP** | **super-controls** | **unilateral early AMD** | **bilateral early AMD** | **unilateral interm. AMD** | **bilateral interm. AMD** | **unilateral nAMD** | **bilateral nAMD** | **unilateral GA** | **bilateral**  **GA** | **late AMD mixed type** |
| --- | --- | --- | --- | --- | --- | --- | --- | --- | --- | --- |
| CFH rs800292 | 1.76, p=0.17 | 1.02, p=0.91 | **0.67, p=0.05** | 1.22, p=0.28 | **0.52, p=0.0002** | **0.43, p=1.53x10^-10^** | **0.35, p=4.28x10^-13^** | 0.69, p=0.29 | **0.39, p=0.002** | **0.37, p=0.004** |
|  | 1.31, p=0.47 | **1.80, p=0.03** | 1.16, p=0.68 | 0.85, p=0.73 | 0.62, p=0.21 | **0.45, p=0.01** | **0.24, p=0.0003** | 0.97, p=0.97 | 0.24, p=0.24 | n/a |
| CFH rs12144939 | 0.73, p=0.38 | 0.94, p=0.68 | **0.64, p=0.03** | **0.60, p=0.02** | **0.29, p=8.43x10^-9^** | **0.41, p=2.23x10^-10^** | **0.30, p=1.44x10^-13^** | **0.33, p=0.009** | **0.24, p=0.0001** | **0.30, p=0.002** |
|  | 0.75, p=0.45 | 0.73, p=0.41 | 0.39. p=0.12 | 0.54, p=0.25 | 0.59, p=0.06 | **0.30, p=0.003** | **0.15, p=0.0001** | n/a | 0.17, p=0.09 | 0.17, p=0.10 |
| C3 rs1047286 | 0.63, p=0.37 | 1.08, p=0.64 | 1.01, p=0.67 | 0.89, p=0.62 | 1.42, p=0.07 | 0.95, p=0.71 | **1.36, p=0.05** | 1.09, p=0.86 | 1.33, p=0.46 | 1.91. p=0.06 |
|  | 0.64, p=0.39 | 1.85, p=0.10 | 1.47, p=0.48 | **3.76, p=0.001** | **2.85, p=0.01** | 1.90, p=0.06 | **3.14, p=0.001** | 3.76, p=0.10 | **7.73, p=0.0002** | 3.05, p=0.18 |
| C3 rs2230199 | 0.66, p=0.41 | 1.00, p=1.00 | 1.00, p=0.99 | 0.76, p=0.26 | 1.33, p=0.14 | 0.99, p=0.96 | **1.42, p=0.02** | 1.02, p=0.96 | 1.43, p=0.35 | 1.64, p=0.18 |
|  | 0.64, p=0.39 | **2.39, p=0.008** | 1.32, p=0.61 | **3.60, p=0.001** | **2.60, p=0.02** | 1.68, p=0.13 | **3.21, p=0.0003** | 3.55, p=0.12 | **7.09, p=0.0004** | **8.00, p=0.0005** |
| CFB rs4151667 | n/a | 1.37, p=0.15 | 1.09,p=0.77 | 0.45 p=0.07 | **0.50, p=0.04** | **0.45, p=0.002** | **0.47, p=0.004** | 0.43, p=0.26 | 0.26, p=0.06 | **0.14, p=0.05** |
|  | n/a | n/a | n/a | n/a | n/a | n/a | n/a | n/a | n/a | n/a |
| CFB rs641153 | 4.83,p=0.24 | 1.02, p=0.93 | 0.70, p=0.19 | 0.60, p=0.14 | **0.43, p=0.002** | **0.49, p=0.0002** | **0.42, p=2.86x10^-5^** | 0.92, p=0.85 | **0.27, p=0.01** | **0.23, p=0.02** |
|  | 4.37,p=0.28 | 0.87, p=0.89 | n/a | n/a | n/a | 1.44, p=0.63 | n/a | n/a | n/a | n/a |
| CFI rs10033900 | 1.12,p=0.59 | 0.86, p=0.38 | 0.89, p=0.59 | 0.93, p=0.74 | 0.93, p=0.69 | 0.93, p=0.58 | 0.83, p=0.20 | 2.28, p=0.10 | 1.13, p=0.70 | 1.85, p=0.14 |
|  | 1.04, p=0.86 | 0.78, p=0.23 | 1.22, p=0.42 | 1.24, p=0.41 | 1.17, p=0.46 | 1.00, p=0.98 | 1.16, p=0.37 | 1.99, p=0.27 | 1.29, p=0.58 | 1.55, p=0.37 |
| CETP rs3764261 | 0.87, p=0.57 | 1.12, p=0.45 | 0.95, p=0.79 | 1.27, p=0.23 | 1.24, p=0.19 | 1.25, p=0.07 | **1.51, p=0.001** | 1.30, p=0.44 | 1.48, p=0.14 | 1.15, p=0.66 |
|  | 1.03, p=0.91 | 1.33, p=0.20 | 1.12, p=0.70 | **1.92, p=0.02** | **1.85, p=0.006** | 1.38, p=0.08 | **1.63, p=0.01** | 0.82, p=0.75 | 1.20, p=0.68 | 1.74, p=0.21 |
| TIMP3 rs9621532 | n/a | 0.83, p=0.46 | 0.95, p=0.88 | 0.59, p=0.16 | 1.11, p=0.68 | **0.63, p=0.03** | **0.51, p=0.008** | 0.95, p=0.92 | 1.70, p=0.13 | 0.16, p=0.07 |
|  | n/a | n/a | n/a | n/a | n/a | n/a | 0.41, p=0.44 | n/a | 3.94, p=0.21 | n/a |
| APOE rs2075650 | 1.17, p=0.78 | 1.22, p=0.21 | 1.08, p=0.73 | 0.63, p=0.06 | 0.86, p=0.41 | **0.66, p=0.004** | 0.89, p=0.41 | 0.91, p=0.81 | 0.51, p=0.07 | 0.55, p=0.14 |
|  | 0.98, p=0.97 | 0.86, p=0.81 | 1.02, p=0.98 | 0.48, p=0.48 | 0.66, p=0.58 | 0.42, p=0.16 | 1.88, p=0.11 | n/a | 2.14, p=0.31 | n/a |
| APOE rs4420638 | 1.87, p=0.12 | 0.95, p=0.75 | 0.88, p=0.52 | **0.57, p=0.02** | 0.81, p=0.24 | 0.85, p=0.19 | 0.89, p=0.38 | 0.89, p=0.75 | **0.52, p=0.05** | 0.93, p=0.81 |
|  | 1.48, p=0.44 | 0.56, p=0.28 | 0.48, p=0.31 | 0.48, p=0.32 | 0.69, p=0.48 | **0.39, p=0.05** | 0.99, p=0.99 | 0.93, p=0.95 | 1.46, p=0.55 | 0.74, p=0.77 |
| TGFBR1rs334353 | 0.63, p=0.12 | 1.22, p=0.17 | 1.23, p=0.27 | 1.15, p=0.48 | 0.88, p=0.41 | 1.13, p=0.32 | 0.88, p=0.31 | 1.09, p=0.80 | 0.80, p=0.41 | 1.04, p=0.75 |
|  | 0.59, p=0.09 | 0.99, p=0.97 | 0.54, p=0.20 | 1.19, p=0.62 | 0.52, p=0.08 | 0.67, p=0.12 | **0.52, p=0.03** | 1.09, p=0.89 | 1.17, p=0.74 | 0.24, p=0.17 |
| SKIV2L rs429698 | 1.34,p=0.63 | 1.24, p=0.16 | 0.90, p=0.63 | **0.55, p=0.02** | **0.44, p=0.0002** | **0.48, p=3.21x10^-6^** | **0.49, p=1.28^-5^** | 0.81, p=0.59 | **0.32, p=0.004** | **0.21. p=0.002** |
|  | 1.09, p=0.89 | 0.76, p=0.66 | 0.79, p=0.75 | 1.10, p=0.88 | 0.47, p=0.30 | 0.55, p=0.55 | **0.11, p=0.01** | n/a | n/a | n/a |
| VEGFA rs943080 | 0.89, p=0.61 | 1.09, p=0.64 | 1.19, p=0.47 | **0.62, p=0.02** | **1.58, p=0.03** | 1.04, p=0.78 | 1.19, p=0.27 | 1.26, p=0.56 | 1.55, p=0.21 | 1.16, p=0.70 |
|  | 1.11, p=0.60 | 1.05, p=0.79 | 1.35, p=0.24 | 0.63, p=0.06 | **1.66, p=0.03** | 1.28, p=0.12 | **1.51, p=0.02** | 0.66, p=0.43 | 1.95, p=0.08 | 1.95, p=0.10 |
| RAD51B rs8017304 | 1.13, p=0.59 | 1.16, p=0.33 | 1.00, p=0.99 | 1.02, p=0.93 | 0.85, p=0.34 | 0.96, p=0.72 | 0.83, p=0.15 | 1.28, p=0.47 | 0.78, p=0.35 | 0.64, p=0.16 |
|  | 0.94, p=0.80 | 0.77, p=0.25 | 0.96, p=0.88 | 0.90, p=0.72 | 1.01, p=0.97 | **0.65, p=0.02** | **0.43, p=7.54x10^-5^** | 0.60, p=0.37 | 0.75 , p=0.44 | 0.80, p=0.59 |
| TNFRSF10A rs1327806 | 1.34, p=0.17 | 1.20, p=0.32 | 1.11, p=0.64 | 1.17, p=0.52 | **1.57, p=0.03** | **1.48, p=0.009** | **1.47, p=0.01** | 0.50, p=0.06 | 1.50, p=0.27 | 1.23, p=0.63 |
|  | 1.12, p=0.54 | 1.32, p=0.16 | 0.91, p=0.71 | 1.45, p=0.15 | **1.56, p=0.05** | **1.44, p=0.03** | **1.49, p=0.02** | 0.45, p=0.09 | **2.74, p=0.007** | **3.49, p=0.003** |

First line: heterozygous variant, second line: homozygous variant showing odds ratio and p-value. Significant associations marked in bold, analysis adjusted for age, reference: no AMD; SNPs = single nucleotide polymorphisms, AMD = age-related macular degeneration, interm. = intermediate, nAMD = neovascular AMD, GA = geographic atrophy.
