# Supplementary material for: Genetics of Unilateral and Bilateral Age-Related Macular Degeneration Severity Stages
Source: PLoS One. 2016 Jun 3;11(6):e0156778. doi: 10.1371/journal.pone.0156778 (PMC4892556; doi:10.1371/journal.pone.0156778)
Supplement: S3 Table — (DOCX) [file pone.0156778.s003.docx]

S3 Table: Multivariate regression model of different AMD severity stages

| **SNP** | **unilateral early AMD** | **bilateral early AMD** | **unilateral interm. AMD** | **bilateral interm. AMD** | **unilateral nAMD** | **bilateral nAMD** | **unilateral GA** | **bilateral**  **GA** | **late AMD mixed type** |
| --- | --- | --- | --- | --- | --- | --- | --- | --- | --- |
| CFH rs800292 | 1.28 (0.91-1.79) | 0.91 (0.58-1.43) | 1.10 (0.69-1.76) | **0.60 (0.39-0.95)** | **0.56 (0.38-0.81)** | **0.36 (0.23-0.56)** | 0.32 (0.10-1.08) | 0.42 (0.16-1.13) | **0.28 (0.08-0.94)** |
|  | p=0.16 | p=0.69 | p=0.68 | **p=0.03** | **p=0.002** | **p=6.28x10^-6^** | p=0.07 | p=0.09 | **p=0.04** |
| CFH rs12144939 | 1.16 (0.81-1.68) | 0.74 (0.45-1.22) | 0.67 (0.40-1.12) | **0.29 (0.16-0.50)** | **0.47 (0.31-0.70)** | **0.34 (0.21-0.54)** | **0.24 (0.06-0.94)** | **0.09 (0.02-0.48)** | **0.27 (0.09-0.87)** |
|  | p=0.42 | p=0.24 | p=0.12 | **p=8.76x10^-6^** | **p=0.0002** | **p=5.92x10^-6^** | **p=0.04** | **p=0.003** | **p=0.03** |
| C3 rs1047286 | 1.01 (0.52-1.99) | 1.42 (0.61-3.34) | 1.70 (0.69-4.18) | 1.86 (0.81-4.28) | 1.02 (0.54-1.93) | 0.98 (0.48-2.03) | 1.45 (0.22-9.54) | 1.45 (0.37-5.66) | 0.38 (0.09-1.57) |
|  | p=0.96 | p=0.41 | p=0.25 | p=0.14 | p=0.96 | p=0.97 | p=0.70 | p=0.59 | p=0.18 |
| C3 rs2230199 | 1.26 (0.65-2.44) | 0.70 (0.30-1.63) | 0.73 (0.30-1.80) | 0.85 (0.37-1.95) | 1.07 (0.56-2.03) | 1.44 (0.70-2.94) | 0.99 (0.15-6.38) | 1.13 (0.27-4.71) | **4.50 (1.15-17.56)** |
|  | p=0.49 | p=0.40 | p=0.49 | p=0.70 | p=0.85 | p=0.32 | p=0.99 | p=0.86 | **p=0.03** |
| CFB rs4151667 | 0.81 (0.27-2.47) | 0.93 (0.21-4.18) | **0.16 (0.04-0.63)** | 0.56 (0.10-3.14) | 0.88 (0.27-2.85) | 0.27 (0.06-1.19) | 0.14 (0.01-1.99) | 0.15 (0.02-1.51) | 0.10 (0.00-4.87) |
|  | p=0.71 | p=0.92 | **p=0.009** | p=0.51 | p=0.84 | p=0.08 | p=0.15 | p=0.11 | p=0.25 |
| CFB rs641153 | 0.98 (0.35-2.75) | 0.64 (0.15-2.74) | **0.23 (0.07-0.72)** | 0.67 (0.14-3.20) | 0.56 (0.19-1.66) | 0.37 (0.10-1.39) | 0.37 (0.04-3.48) | **0.03 (0.00-0.38)** | 0.33 (0.01-8.60) |
|  | p=0.97 | p=0.55 | **p=0.01** | p=0.61 | p=0.30 | p=0.14 | p=0.38 | **p=0.006** | p=0.50 |
| CFI rs10033900 | 0.87 (0.69-1.10) | 1.23 (0.91-1.66) | 1.21 (0.89-1.65) | 1.05 (0.79-1.39) | 1.01 (0.80-1.26) | 1.17 (0.90-1.52) | 1.20 (0.58-2.46) | 0.98 (0.53-1.81) | 1.66 (0.87-3.17) |
|  | p=0.26 | p=0.19 | p=0.22 | p=0.75 | p=0.97 | p=0.25 | p=0.62 | p=0.95 | p=0.13 |
| CETP rs3764261 | 1.18 (0.94-1.49) | 1.12 (0.83-1.52) | **1.49 (1.10-2.01)** | **1.40 (1.06-1.85)** | **1.28 (1.03-1.60)** | **1.31 (1.01-1.71)** | 1.61 (0.79-3.30) | 1.11 (0.60-2.03) | 1.27 (0.69-2.34) |
|  | p=0.16 | p=0.46 | **p=0.01** | **p=0.02** | **p=0.03** | **p=0.04** | p=0.19 | p=0.74 | p=0.44 |
| TIMP3 rs9621532 | 0,73 (0.42-1.30) | 0.64 (0.30-1.40) | 0.46 (0.19-1.08) | 0.94 (0.51-1.73) | 0.57 (0.32-1.01) | **0.49 (0.25-0.98)** | 0.32 (0.05-2.04) | **3.15 (1.34-7.41)** | 0.23 (0.03-2.14) |
|  | p=0.29 | p=0.26 | p=0.07 | p=0.84 | p=0.06 | **p=0.04** | p=0.23 | **p=0.008** | p=0.20 |
| APOE rs2075650 | **1.71 (1.14-2.57)** | 1.38 (0.80-2.35) | 0.87 (0.46-1.63) | 1.05 (0.60-1.85) | 0.79 (0.52-1.22) | 1.08 (0.65-1.80) | 1.13 (0.25-5.15) | 0.77 (0.22-2.69) | 0.72 (0.21-2.44) |
|  | **p=0.01** | p=0.25 | p=0.65 | p=0.86 | p=0.29 | p=0.77 | p=0.87 | p=0.69 | p=0.60 |
| APOE rs4420638 | 0.70 (0.47-1.05) | 0.76 (0.46-1.28) | 0.68 (0.39-1.18) | 0.84 (0.50-1.39) | 0.91 (0.63-1.32) | 0.85 (0.54-1.34) | 0.56 (0.15-2.06) | 0.69 (0.26-1.83) | 0.71 (0.27-1.84) |
|  | p=0.89 | p=0.30 | p=0.17 | p=0.49 | p=0.62 | p=0.49 | p=0.38 | p=0.45 | p=0.48 |
| TGFBR1rs334353 | 1.22 (0.94-1.57) | 0.83 (0.58-1.18) | 1.18 (0.85-1.65) | **0.69 (0.49-0.98)** | 0.82 (0.63-1.06) | 0.76 (0.56-1.03) | 1.05 (0.49-2.23) | 0.70 (0.35-1.40) | 0.57 (0.26-1.24) |
|  | p=0.13 | p=0.29 | p=0.33 | **p=0.04** | p=0.13 | p=0.08 | p=0.91 | p=0.31 | p=0.15 |
| SKIV2L rs429698 | 1.19 (0.44-3.22) | 1.13 (0.29-4.44) | 2.28 (0.90-5.75) | 0.66 (0.15-3.00) | 0.95 (0.34-2.66) | 1.32 (0.38-4.60) | 4.00 (0.49-32.88) | 4.34 (0.76-24.88) | 0.63 (0.04-11.26) |
|  | p=0.73 | p=0.87 | p=0.08 | p=0.61 | p=0.92 | p=0.66 | p=0.20 | p=0.10 | p=0.75 |
| VEGFA rs943080 | 1.03 (0.82-1.30) | 1.07 (0.80-1.44) | 0.86 (0.64-1.16) | 1.24 (0.93-1.65) | 1.24 (0.99-1.54) | 1.27 (0.99-1.63) | 0.57 (0.28-1.16) | 0.95 (0.54-1.68) | 1.24 (0.66-2.24) |
|  | p=0.80 | p=0.64 | p=0.32 | p=0.42 | p=0.06 | p=0.06 | p=0.12 | p=0.86 | p=0.48 |
| RAD51B rs8017304 | 0.99 (0.78-1.25) | 0.90 (0.67-1.22) | 0.96 (0.70-1.31) | 0.97 (0.73-1.28) | 0.83 (0.67-1.04) | **0.71 (0.54-0.94)** | 0.81 (0.40-1.61) | 0.58 (0.31-1.06) | 0.77 (0.42-1.41) |
|  | p=0.93 | p=0.50 | p=0.78 | p=0.81 | p=0.11 | **p=0.02** | p=0.54 | p=0.08 | p=0.39 |
| TNFRSF10A rs1327806 | 1.10 (0.88-1.38) | 1.00 (0.74-1.33) | 1.07 (0.80-1.44) | 1.22 (0.92-1.61) | 1.12 (0.90-1.39) | 1.23 (0.95-1.59) | 0.62 (0.32-1.21) | **2.98 (1.59-5.60)** | **2.51 (1.35-4.65)** |
|  | p=0.40 | p=0.94 | p=0.66 | p=0.17 | p=0.32 | p=0.12 | p=0.16 | **p=0.001** | **p=0.004** |
| Age | **1.04 (1.02-1.06)** | **1.06 (1.04-1.09)** | **1.11 (1.08-1.14)** | **1.12 (1.10-1.15)** | **1.12 (1.10-1.15)** | **1.20 (1.17-1.23)** | **1.17 (1.10-1.23)** | **1.19 (1.13-1.25)** | **1.26 (1.19-1.34)** |
|  | **p=0.0001** | **p=2.27x10^-6^** | **p=9.02x10^-15^** | **p=5.40x10^-21^** | **p=6.58x10^-29^** | **p=1.17x10^-47^** | **p=4.01x10^-8^** | **p=1.12x10^-11^** | **p=5.26x10^-15^** |

First line showing odds ratio and 95% Confidence interval, second line showing p-value. Significant associations marked in bold, reference: no AMD; SNPs = single nucleotide polymorphisms, AMD = age-related macular degeneration, interm. = intermediate, nAMD = neovascular AMD, GA = geographic atrophy.
